# Multimodal Advertisement of Pregnancy in Free-Ranging Female Japanese Macaques (*Macaca fuscata*)

**DOI:** 10.1371/journal.pone.0135127

**Published:** 2015-08-26

**Authors:** Lucie Rigaill, Andrew J. J. MacIntosh, James P. Higham, Sandra Winters, Keiko Shimizu, Keiko Mouri, Takeshi Furuichi, Cécile Garcia

**Affiliations:** 1 Social Systems Evolution Section, Department of Ecology and Social Behaviors, Primate Research Institute, Kyoto University, Inuyama, Japan; 2 Wildlife Research Center, Kyoto University, Kyoto, Japan; 3 Department of Anthropology, Center for the Study of Human Origins, New York University, New York, United States of America; 4 Department of Zoology, Faculty of Science, Okayama University of Science, Okayama city, Okayama, Japan; 5 Laboratoire de Dynamique de l’Evolution Humaine, UPR 2147, CNRS, Paris, France; University of Pisa, ITALY

## Abstract

The role of multiple sexual signals in indicating the timing of female ovulation, and discrimination of this timing by males, has been particularly well studied among primates. However the exhibition of pregnancy signals, and how such signals might modulate male post-conception mating decisions, is still poorly understood. Here we aimed to determine if Japanese macaque males use changes in female sexual signals (behavioral, visual and auditory) to discriminate pregnancy and adjust their socio-sexual behaviors. We combined behavioral observations, digital photography and endocrinological (progestogen and estrogen) data, collected systematically during three one-month periods: the pre-conceptive period, the 1^st^ month of pregnancy and the 2^nd^ month of pregnancy. We analyzed variation in the probability of detecting male and female socio-sexual behaviors and estrus calls, as well as changes in female face color parameters, in relation to female reproductive state. Based on our focal observations, we found that males did not copulate during the pregnancy period, and that female socio-sexual behaviors generally decreased from the pre-conceptive to post-conceptive periods. Female face luminance decreased from the pre-conceptive month to the pregnancy period whereas face color only varied between the 1^st^ and 2^nd^ month of gestation. Our results suggest that Japanese macaque females display sexual cues of pregnancy that males might use to reduce energy wasted on non-reproductive copulations with pregnant females. We hypothesize that females advertize their pregnancy through changes in behavioral, visual and potential auditory signals that males can use to adjust their mating behaviors. We finish by discussing implications for male and female post-conception strategies.

## Introduction

Primate males usually concentrate their reproductive efforts towards females during the period of highest conception probability, i.e. the peri-ovulatory or fertile phase of the menstrual cycle [1–5]. However, non-reproductive copulations during pregnancy are not unusual among New World (capuchins: [1]) and Old World (sooty mangabeys: [2,] Hanuman langurs: [3], Phayre’s langurs: [4], rhesus macaques: [5], pig-tailed macaques: [6], stumptailed macaques: [7], Japanese macaques: [8], long-tailed macaques: [9], Assamese macaques: [10], Barbary macaques: [11], chimpanzees: [12], bonobos: [13]) primates. However, many of these reports were based on observations of mounts, either with or without intromission and ejaculation, collected unsystematically, with inferences made about female reproductive state in the absence of endocrinological confirmation of pregnancy. The results from studies using systematic methods have produced different conclusions for different species; males of some species seem to discriminate pregnancy from the pre-conceptive period to some extent (Assamese macaques: [10], langurs: [4, 14]), whereas in other species pregnancy might be concealed (long-tailed macaques: [9], Barbary macaques: [11]).

Several hypotheses have been suggested to explain the occurrence and the role of these copulatory behaviors during non-reproductive periods. Post-conceptive sexual behaviors might: 1) be an incidental by-product of changes in female sexual hormones (estrogens and progesterone) throughout pregnancy [9, 14]; 2) be a female strategy used to confuse paternity and thus decrease the risk of potential infanticide by males [9]; 3) allow both males and females to develop or maintain close relationships with preferred and/or new partners in order to improve future long-term reproductive success [8, 10], and; 4) help the formation of social bonds between females especially in the case of newly immigrant females (see bonobos: [15, 16]). However, without a better understanding of the potential communication of pregnancy status and the potential causes of post-conception mating (i.e. variations in female sexual signals between pre- and post-conception, and their use by males to discriminate the female reproductive state), drawing clear conclusions is impossible.

Since mating can be very costly to females (e.g. exhibition of sexual swellings [17]), and males (e.g. mate guarding [18]), there should have been selection for the ability to signal (females) and discriminate (males) pregnancy in order not to waste energy on non-reproductive mating. Previous studies have suggested that female behavioral (e.g. approaches and presentations: [19–22]), auditory (e.g. copulation calls: [23–25]), visual (e.g. sexual swelling size and face or sexual skin color: [26–29]) and olfactory (i.e. female vaginal and urinary compounds: [30–32]) signals can allow males to discriminate between females (cycling vs. non-cycling) and within females (i.e. the fertile phase and the timing of ovulation vs. the non-fertile phases). Yet little is known about the changes in female sexual signals as indicators of early pregnancy, with most studies focusing only on female solicitations (behavioral signals) or post-conceptive swelling size (visual signals). Indeed, it has been reported that some non-human primate females exhibit proceptive behaviors during their pregnancy (bonobos: [15, 16], langurs: [3, 33], Barbary macaques: [34], Japanese macaques: [35]), and in some species, sexual swellings have been observed during pregnancy (bonobos: [13], sooty mangabeys: [2], long-tailed macaques: [9, 36], Barbary macaques: [11]). In the two macaque species studied, the swelling did not differ in size [9] or in the duration of the maximal size [11] between pre- and post-conceptive phases. As a result, it seems clear that, for some primate species, female sexual signals are present after conception, and thus conceal early pregnancy to males. Other species however, might signal their pregnancy through changes in female sexual signals, allowing males to discriminate between pre- and post-conceptive periods.

Japanese macaques are usually described as a species in which mating behaviors are not restricted solely to the timing of ovulation and for which copulations during pregnancy are not rare [8, 35, 37–39] (but see: [40]). As this species lacks sexual swellings, females seem to advertize their fertility status through several other sexual signals. Some female sexual behaviors (e.g. mounts, holding behaviors) and auditory signals (i.e. copulation and estrus calls) might indicate the follicular phase [41], but not accurately pinpoint the fertile phase [42], and females are usually described as “redder” around their so-called “estrus phase” [40]. However, to our knowledge, no studies of Japanese macaques have analyzed the occurrence of post-conceptive sexual behaviors in a systematic way (i.e. through focal observations and statistical analyses) by assessing the impact of female sexual signals (behavioral, visual and auditory) on male and female post-conceptive mating strategies.

The aims of our study were therefore to: (1) assess variation in female sexual hormones (progestogen and estrogen) during pregnancy; (2) analyze changes in female socio-sexual behaviors between pre- and post-conceptive phases; (3) investigate potential variation in female visual (face color and luminance) and auditory (estrus calls) sexual signals between pre- and post-conceptive phases that might influence males in their mating strategies; and ultimately (4) investigate male behavioral responses to changes in female sexual signals between pre- and post-conceptive periods. By doing so, this study aims to determine the underlying mechanisms involved in multimodal pregnancy signaling, and to assess its potential adaptive significance.

## Methods

This study was carried out in the field with free-ranging monkeys and was completely non-invasive. This research protocol was reviewed and approved by the Wildlife Research Center of Kyoto University and was in agreement with the Guidelines for the Care and Use of Nonhuman Primates of the Kyoto University Primate Research Institute.

### Study site and subjects

Koshima is a 0.32 km² uninhabited mountainous islet located in Miyazaki prefecture (31°220 N, 131°260 E), Kyushu, Japan. At Koshima, females mate from early December to mid March and deliver mainly between June and September. Koshima macaques are very well habituated to humans and easily identifiable through facial marks, both artificial (tattoos) and natural. We conducted our study from December 2013 to mid-March 2014 following the main troop of Koshima macaques which was composed of 64 individuals: 20 adult (i.e. ≥ 6 years old) females (14.2 ± 3.2 years old, range = 8–18), 7 adult males, 29 subadult males and females, and 8 infants (less than 1 year old). For this study, we first identified all adult females that had the potential to resume cycling in this year’s mating season (i.e. females older than 6 years old and/or with an infant of more than 2 years-old). Two of these females were excluded because we did not want their involvement in a separate parasite removal experiment led by AJJM to bias our results, resulting in a list of ten focal females. Three females of our sample did not resume cycling during the mating season and were therefore excluded from the analyses. Of the seven females observed to cycle, five conceived during the study period (mean = 11.6 ± SD 3.8 years, range = 8–17) and were used in our analyses. Although this represents a small sample size, it is worth bearing in mind that no comparable data are available for wild Japanese macaques. Group composition remained unchanged during the study period.

### Behavioral observations

Behavioral data were collected by one observer (LR) using focal animal sampling [43] distributed from 8.00 AM to 4.00 PM, 7 days per week, yielding a total of 454 hours of observation over the 3.5 month period. We maximized our efforts by following each female 2 hours per week, with 6 blocks of 20-min continuous focal sampling equally distributed across days and females. Due to exceptionally bad weather conditions we were sometimes unable to access the island, with the biggest gap occurring in February 2014. We selected 153 focal observations for analyses: 46 for the month preceding the pregnancy (i.e. pre-conceptive period or PCP, mean per female = 9.2, range = 3–14 focal), 57 for the first month of pregnancy (mean per female = 11.4, range = 9–15 focal) and 50 for the second month (mean per female = 10.0, range = 9–11) (see below for calculations of pregnancy length and conception dates).

During focal observations, we recorded all social and sexual behaviors between males and females, along with the direction of behavior between the focal female and any adult male, and the identity of the other individual involved. For the social interactions, we recorded the number of grooming bouts between males and females along with the time spent grooming and being groomed. The number of contacts made or broken between males and females, the total duration of contact bouts, and the identity of the individual engaged in contact were also recorded continuously during the focal observations. Female proceptivity, or motivation to copulate [44], was assessed by monitoring female sexual behaviors, e.g. approaches, sexual presentations, and holding behaviors. We also recorded the occurrence of female estrus calls and copulation calls [23] during mounting series (see definitions and distribution in Table 1). Male sexual behaviors directed to females were used to assess male response to female sexual signals. We recorded male approaches, holding behaviors, olfactory (nasal) inspections of the anogenital area [45], non-ejaculatory and ejaculatory mounts (Table 1). Female dominance ranks were monitored using focal and *ad libitum* observations and were assessed by transcribing submissive (avoidances, retreats) and aggressive behaviors (attacks, threats and chases) into an agonistic interaction matrix. We calculated the Normalized David’s Score (NDS) to assess female rank positions following previously described methods [46, 47].

**Table 1:** Definition and distribution of female and male sexual behaviors, female estrus and copulation calls in relation to female reproductive status (i.e. pre-conceptive period PCP, first and second months of pregnancy).

Sexual behaviors	Definitions	PCP	First month	Second month
Female approaches	Female comes close (less than 1m) or in contact with a male	130	21	82
Female sexual presentations	Female directs her anogenital area toward a male	0	0	0
Female holding behaviors	Female is sitting behind the male and grabs his back/torso, generally observed just before mating	0	0	27
Estrus calls	Female utters loud and long calls when alone or in the proximity of a male	1809	804	3065
Copulation calls	Female utters special vocalizations during the last stage of the mount	57	0	0
Male approaches	Male comes close (less than 1m) or in contact with a female	137	6	27
Male holding behaviors	Male grabs female hips, generally observed just before mating	163	0	45
Mounts	Male mounts a female, with intromission of the penis and ejaculation if successful (ejaculatory) or not (non-ejaculatory)	138	0	0
Olfactory inspections	Deliberate placing of the nostrils to within 5 cm of a female’s anogenital area	0	0	0

### Assessment of female face color and luminance

**Fig 1:**
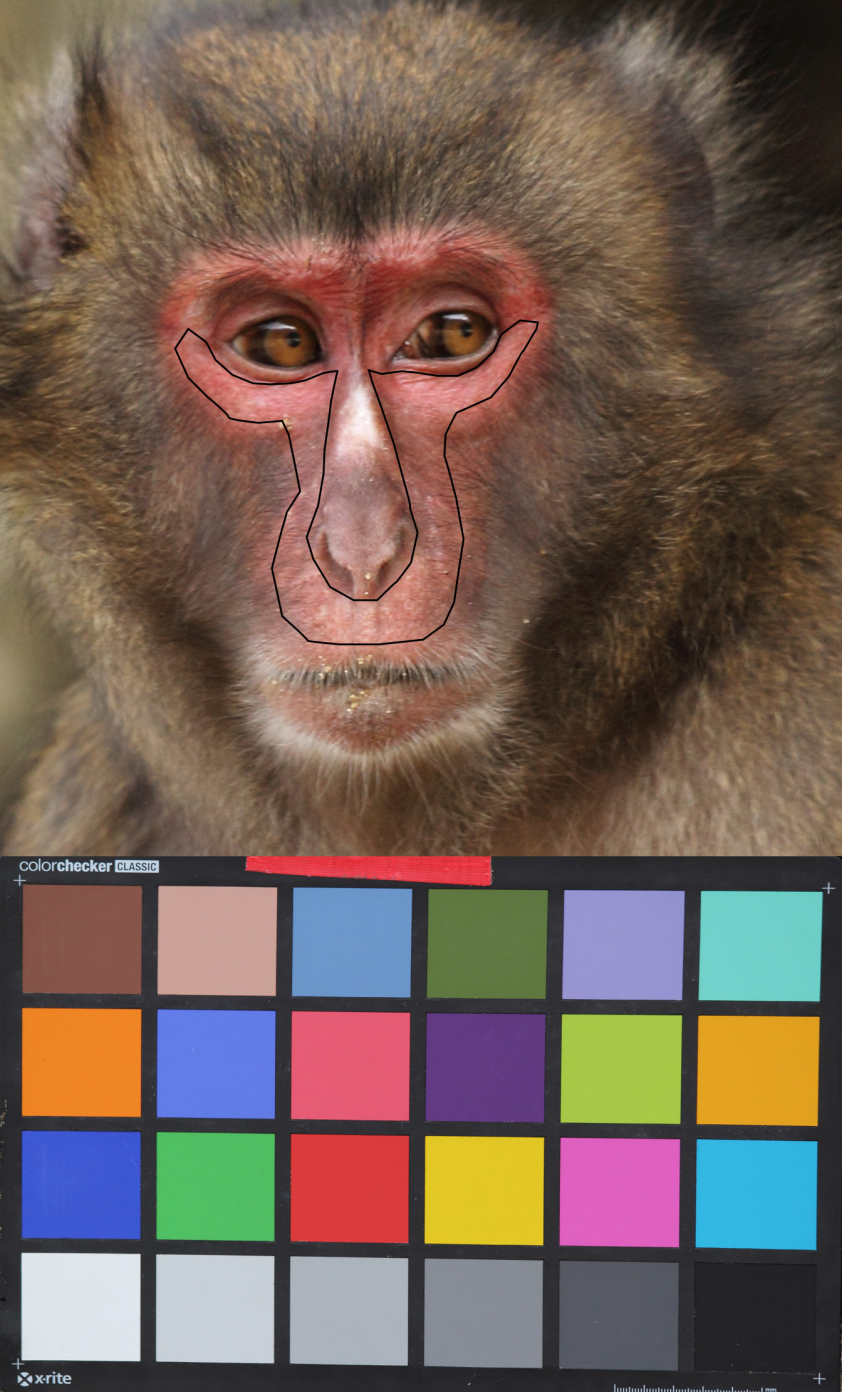
Picture of a female face with Gretag color standard showing the area used for color analyses.

Objective collection of face redness (chromatic) and luminance (achromatic) data followed the method previously described in Rigaill et al. [29]. Photos were usually taken in the morning between 8.00 AM and 12.00 PM, whenever possible in an open area of the island where the light conditions were optimal, rather than in the forest. We used a Canon EOS 350D camera with an 8 megapixel CMOS censor and an EF 28–135 mm f/3.5–5.6 IS USM lens. White balance was set manually using an X-Rite White Balance Card (GretagMacbeth ColorChecker) before daily data collection, and reset between individual photo captures if needed (e.g. when the time lag between each individual’s photo was too long or when the light conditions changed drastically). We collected images in RAW format with the flash disabled and with the shutter speed and aperture size determined automatically by the camera. To standardize images of face color, we used a Gretag X-Rite Color Checker (24 colored squares of known and varying reflectance) placed on an easel 5 cm off the ground. An image of this chart was taken immediately after the female face image. We placed the chart at the exact same position (i.e. distance and orientation) from the camera as the subject with camera settings standardized such that the image of the female face and the corresponding image of the chart was captured using the same camera settings (shutter speed and aperture size) [28, 29, 48]. This technique allowed images to be standardized such that color measurements were comparable across all images. We used a total of 88 photos for color analyses (PCP: N = 28, mean per female = 5.6 ± SD 1.9 photos; 1^st^ month: N = 35, mean per female = 7.0 ± SD 0.7 photos; 2^nd^ month: N = 25, mean per female = 5.0 ± SD 1.9 photos). We assessed color from the whole face excluding the eyes, nose, front and identity tattoos (Fig 1) following previously described methods using Colourworker software [29, 49]. We converted our camera sensor measures to estimates of Japanese macaque retinal receptor stimulation using previously published methods [29, 49, 50]. We calculated the red–green opponency channel (i.e. R/G ratio) as (LW-MW)/(LW+MW) to assess how female face varied chromatically at long-wavelengths, and we also analyzed the facial luminance as (MW+LW/2) to measure how light or dark the face was according to our model of Japanese macaque visual perception.

### Fecal sample collection and hormonal analyses

During the study period, we collected 138 fecal samples from 7 focal females (mean per females = 14.3 ± SD 1.8 fecal samples, range = 13–18). Fecal collection was carried out during the morning focal observations between 8.00 AM and 12.00 PM, as well as opportunistically. Samples were collected in their entirety, bagged and labeled immediately after voiding. Approximately 2g of all samples were then stored in 50 mL plastic tubes, with 35 mL of silica gel added to allow a complete desiccation of the pellet. Fecal samples were then analyzed for estrone conjugates (E1C) and pregnanediol-3-glucuronide (PdG) using previously described and validated enzyme immunoassays (EIA) (for further details see [42, 51]), with a few modifications. Dry feces (0.25 g) were shaken with 2.5 mL of modified phosphate buffer (0.1 M, pH 7.0, 0.1% BSA with 0.05% tween-20) at room temperature for 24 hr, and then centrifuged (3,000 rpm, 10 min). The supernatant was decanted into clean tubes and the residual pellet was discarded. 50 μL of solubilized fecal samples were used for PdG and E1C EIA, using pregnanediol-3-glucuronide-BSA antibody and estrone-3-glucuronide-BSA antibody (Cosmo, FKA334E and FKA224E, Japan). The PdG and E1C concentrations for each sample were calculated by fitting the absorbance of the standard curve using a curve-fitting program (LS-PLATE manager 2004, Wako Pure Chemical Industries, Japan). The E1C assay had a sensitivity of 0.54–7.00 ng/g with an intra-assay coefficient of variation (CV) of 2.15% and inter-assay CV of 4.27%. The PdG assay had a sensitivity of 0.3–6.1 μg/g with an intra-assay CV of 5.43% and inter-assay CV of 4.27%.

### Determination of the conception date and pregnancy period

The presumed days of ovulation and conception were defined using PdG profiles. Following previous studies [42, 52, 53], the onset of the luteal phase was determined as the sample with a fecal PdG concentration which was at least two standard deviations greater than the mean PdG concentration of the 3–4 preceding baseline values. Because of the time lag between circulating hormones and the fecal rise in PdG concentration (2–3 days, see [54]), we determined a 2-day peri-ovulatory period as days -2 and -3 relative to the fecal PdG rise. The fertile phase was then defined as the 5-day period covering these 2 days plus the three preceding days in order to account for the life span of sperm in the female tract (humans: [55]).

Conception can occur at any time during the fertile phase. We considered the latest possible day of fertilization, i.e. the last day of the fertile phase, as the onset of pregnancy. Hormonal data and data on female delivery dates were combined to determine the most likely fertilization date for each focal female, given that gestation in Japanese macaques averages 176.3 days [40, 54]. In total, 59 samples collected during the pregnancy period were used for statistical analyses (1^st^ month: N = 32, mean per female = 6.4 ± SD 2.2 samples; 2^nd^ month: N = 27, mean per female = 5.4 ± SD 2.3 samples; range = 3–9 samples per month, N = 5 females).

### Statistical analyses

Our statistical approach was designed to test whether reproductive status in female macaques was associated with: (i) the performance of socio-sexual behaviors; (ii) female estrus calls, face color and luminance; and (iii) variation in reproductive hormones. We therefore constructed a series of general linear mixed-effects (LME) and generalized linear mixed-effects (GLMM) models fitted by maximum likelihood to examine our data. Each model included reproductive status as a predictor variable with three levels (pre-conceptive period, 1^st^ month of pregnancy, 2^nd^ month of pregnancy) and age as a potentially confounding covariate. Dominance rank is another potentially confounding variable but because of our small data set age and rank were spuriously correlated (r = 1.00), precluding our ability to separate the effects of these variables. Also due to the small sample size, we could not investigate the effect of female reproductive history (e.g. parity) on sexual signaling or male behavioral responses. We accounted for temporal variation and pseudo-replication in each model by setting the date on which the focal sample was collected and the focal individual identity as crossed random effects. We estimated the error structure of each response variable (variables explained below) by comparing the fits of various theoretical cumulative density functions (e.g. normal versus lognormal for continuous distributions, Poisson versus negative binomial for discrete distributions) against our observed data and then ensured that all relevant model assumptions were met (e.g. normality for Gaussian models and homogeneity of residuals for all models) by visually inspecting histograms of the residuals and plots of the residuals against fitted values. Each fitted model was then compared via a likelihood ratio test (LRT) with a null model in which reproductive status had been removed; age was retained in all null models so that these tests focused specifically on the impact of adding or removing the reproductive status term. Any full model that did not significantly outperform its respective null model was discarded and we do not report further on its parameter estimates. Finally, because this statistical approach forces comparisons of all categorical predictor levels against a baseline intercept term (i.e. the pre-conception period for pregnancy status) this factor was re-leveled once so that comparisons between pregnancy periods could also be made. This approach had no effect on other model parameters, including tests of full versus null models.

To determine whether reproductive status was related to female-male socio-sexual behaviors, we were interested in three aspects of each behavior: (i) whether or not the behavior was observed during a focal sample (presence/absence), (ii) the duration (states) or number of occurrences (events) of the behavior during a focal sample; and (iii) the direction of the behavior, where relevant. All behaviors were extracted from focal samples, making the latter the unit of the analysis here. Note that some behaviors could not be analyzed due to infrequency of occurrence during focal observations (Table 1), either overall (female presentations and male olfactory inspections) or in association with specific reproductive status (female holding behaviors, copulations calls, male approaches and mounts). We then created a series of 2x2 contingency tables incorporating all possible combinations of behaviors in binary (presence/absence per focal sample) and count forms, and examined their respective phi (for binary variables) and Pearson’s correlation coefficients. This procedure identified a strong correlation between grooming and body contact (rho = 0.77, which explains ca. 70% of the variance in the data), so we used grooming in our statistical analyses as representative of the major component of contact behavior. Other correlations also existed, e.g. between female approaches and grooming (phi = 0.54), and female approaches and contact (phi = 0.61), but these remain in our analyses below as they are not implicit components of one another in the same way that contact is for grooming.

Thus, in our statistical models, we examined female approaches, female-directed and male-directed grooming bouts, and male holding behavior observed during each focal sample. We constructed hurdle models for each behavior in which a binary (presence/absence) model was first fit before subsequently modeling the zero-truncated (positive values only) count portion of the data [56]. The reason for focusing on the binary component of the data rests with the large number of zeroes observed: the number of 0 values observed during focal samples for all behaviors examined was ≥ 70%. Thus, much of the variance in behaviors could be encapsulated by this simple binary model. We used the package glmmADMB [57, 58] in R to fit these hurdle models in a series of GLMMs with binomial (for binary models) and truncated negative binomial (for count models) error structures. We also tested whether a zero-inflation parameter was necessary in the binary models to account for the large numbers of zeroes observed for all behavior types, but in all cases the simpler binomial model prevailed. The relationship between female estrus calls and reproductive status was examined using the same method.

We tested whether face color and luminance were associated with reproductive status via LMEs with either luminance or R/G ratio as the response variable. Inspection of the cumulative distribution functions (CDFs) of each revealed good fits to the lognormal distribution, so each was log-transformed prior to model fitting. Gaussian models were then fit using the package nlme in R [59]. The relationships between female reproductive hormones and pregnancy status were similarly examined via LMEs, with E1C, PdG and the E1C/PdG ratio used as response variables. Again, inspection of the CDFs of each revealed good fits to the lognormal distribution, so each was log-transformed prior to model fitting and Gaussian models were then fit using nlme. Due to a small sample size during the pre-conceptive month, relationships between estrus calls, sexual and social behaviors and female sexual steroids could not be assessed.

## Results

Full statistical results are available as supplementary material (Tables A-D in S1 File).

### Female sexual hormones during pregnancy

The E_1_C/PdG ratio depended on pregnancy status (LRT: X^2^ = 11.59,Δdf = 4, P < 0.001, see Table A in S1 File for further details), increasing significantly during the second month of pregnancy compared to the first (GLMM: t = 3.49, P < 0.001, see Table B in S1 File for further details). Further analyses showed that while progestogen concentrations were significantly different according to reproductive status (LRT: X^2^ = 7.78, Δdf = 4, P = 0.006), decreasing from the first month of pregnancy to the second month (GLMM: t = -2.06, P = 0.045), concentrations of E_1_C did not differ (LRT: X^2^ = 1.71, Δdf = 2, P = 0.43).

### Variation in female socio-sexual behaviors between pre- and post-conceptive periods

**Fig 2:**
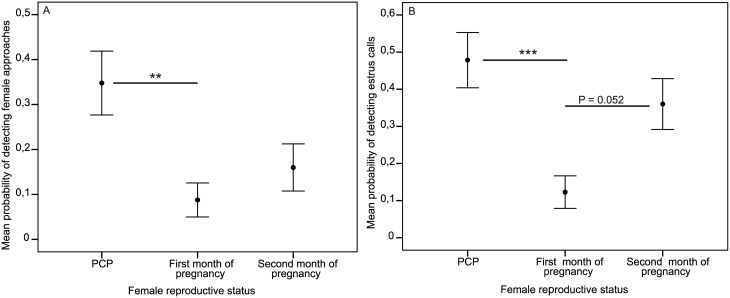
Female behavioral and auditory signals between pre- and post-conceptive periods. Values show mean probability (± standard error of the mean) of detecting (A) female approaches, and (B) estrus calls (* P < 0.050, ** P < 0.010, *** P < 0.001).

The probability of females approaching males changed significantly with reproductive status (LRT: X^2^ = 10.58, Δdf = 4, P = 0.005, see Table C in S1 File for further details); this behavior significantly decreased from the pre-conceptive period to the 1^st^ month of pregnancy (GLMM: z = -2.75, P = 0.006, Fig 2A) and was marginally lower during the second month of pregnancy than the pre-conceptive period as well (GLMM: z = -1.71, P = 0.086; Fig 2A, see Table D for further details). There was no difference in the probability of female approaches between the first and second months of pregnancy (GLMM: z = 1.14, P = 0.26). The probability of grooming performed by females also changed according to reproductive status (LRT: X^2^ = 6.93, Δdf = 4, P = 0.031), decreasing significantly from the pre-conceptive period to both the first (GLMM: z = -2.07, P = 0.038) and second GLMM: z = -2.10, P = 0.036) months of pregnancy, though no differences were observed between pregnancy months (GLMM: z = -0.18, P = 0.86). None of the truncated count models (i.e. female approaches and grooming bouts) significantly outperformed their respective null models (all LRT: P > 0.05).

### Vocal and color signals

The probability of emitting estrus calls was associated with reproductive status (LRT: X^2^ = 13.33, Δdf = 4, P = 0.001, see Table C in S1 File for further details), decreasing significantly between the pre-conceptive period and the first month of pregnancy (GLMM: z = -3.19, P = 0.001, Fig 2B), and then increasing again to near significance from the first to the second month of pregnancy (GLMM: z = 1.94, P = 0.052, Fig 2B, see Table D in S1 File for further details). There were no differences in probability of occurrence between the pre-conceptive period and the second month of pregnancy (GLMM: z = -0.96, P = 0.34). There were no differences in the count of estrus calls performed according to reproductive status in focal samples in which this behavior was observed to occur (LRT: X^2^ = 0.79, Δdf = 4, P = 0.67).

**Fig 3:**
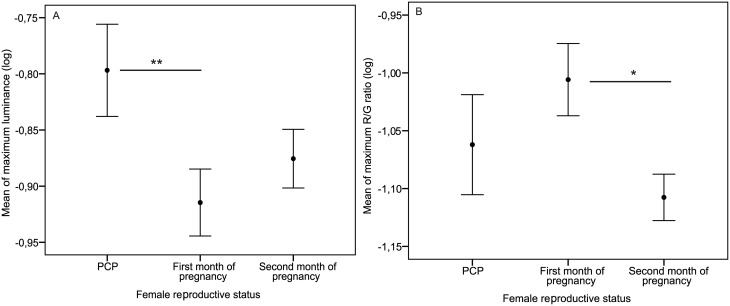
Female facial color features between pre- and post-conceptive phases. Values represent the mean (± standard error of the mean) of maximum luminance (A) and R/G ratio (B) (* P < 0.050, ** P < 0.010, *** P < 0.001).

Female facial features changed according to reproductive status in both luminance (LRT: X^2^ = 7.25, Δdf = 4, P = 0.027, see Table A in S1 File for further details) and R/G ratio (LRT: X^2^ = 4.66, Δdf = 4, P = 0.09). With regards to luminance, faces became significantly darker between the pre-conceptive period and the 1^st^ month of pregnancy (LME: t = -2.66, P = 0.010, Fig 3A), although there were no significant differences between the pre-conceptive period and the second month of pregnancy (LME: t = -1.71, P = 0.092, Fig 3A), or across pregnancy stages (LME: t = 0.77, P = 0.44, see Table B in S1 File for further details). Female faces also became less red during pregnancy with the R/G ratio decreasing significantly between the first and the second month of pregnancy (LME: t = -2.12, P = 0.037, Fig 3B), though this ratio did not vary between the pre-conceptive and either first (LME: t = 1.24, P = 0.22) or second (LME: t = -0.87, P = 0.38) months of pregnancy.

### Male behavioral responses to changes in female sexual signals

We observed too few occurrences of males approaching females during pregnancy for the statistical models to be run. There were also no male holding behaviors observed during the first month of pregnancy, but a comparison of the pre-conceptive period versus the second month of pregnancy revealed an effect near to significance (LRT: X^2^ = 3.45, Δdf = 3, P = 0.063, see Table C in S1 File for further details), with the probability of observing males performing holding behaviors significantly decreasing during the second month of pregnancy (GLMM: z = -2.00, P = 0.045, see Table D in S1 File for further details). Of the 138 copulations recorded during focal observations, none occurred during pregnancy, although 2 mount sequences (ending with ejaculation) were observed for one pregnant female (during the second month of pregnancy) during *ad libitum* observations. The probability of observing male-directed grooming bouts did not significantly differ according to reproductive status (LRT: X^2^ = 2.05, Δdf = 4, P = 0.36). None of the truncated count models (i.e. male holding behaviors and grooming bouts) significantly outperformed their respective null models (all LRT: P > 0.05).

## Discussion

Female Japanese macaques in our study seem to advertize their reproductive state through changes in behavioral and visual signals. Female auditory signals (i.e. estrus calls) might also be involved in pregnancy signaling although more data on the acoustical parameters of calls are needed to fully understand their role given that call rates increased again following the first month of pregnancy. Males seem able to discriminate pregnancy from the pre-conceptive period and refrained from copulating with pregnant females. Such a multimodal framework of pregnancy signaling might have emerged in some populations of Japanese macaques living in harsh environments (i.e. Koshima and Kinkazan populations [40]), where females might need to allocate their energetic resources to fetal growth instead of confusing paternity.

Our most striking results were that males and females did not copulate, displayed fewer sexual behaviors (i.e. approaches and holding behaviors) and females engaged in fewer social interactions with males (e.g. female-directed grooming bouts) during early pregnancy, compared to the month during which conception occurred. This suggests that male Japanese macaques, like some other primate species (Assamese macaques: [10], Hanuman langurs: [14], Sooty mangabeys: [2, 36], and Phayre’s langurs: [4]), but contrary to long-tailed [9] and Barbary [11] macaques, might be able to discriminate the pregnancy period from the cycling phase. Our findings are in agreement with previous reports from Fujita et al [40], but contrast with previous reports on wild and captive Japanese macaques [8, 35, 37, 60, 61], where copulations during pregnancy have been described as not unusual. Although our study was limited by the small sample size, it is important to note that to date no other comparable data on the frequency of copulation between pre- and post-conceptive periods are available for Japanese macaques. Indeed most of the previous studies were conducted in environments that are not representative of natural conditions, did not record variation in sexual behaviors with objective and systematic methods, and did not analyze data statistically; instead, they qualitatively described copulation events during the presumed pregnancy (without distinguishing mounts with or without intromission). We suggest that males Japanese macaques from Koshima (like in the Kinkazan population [40]) might have access to primary information concerning the reproductive stage of the pregnant females, which might influence their mating decisions. Two possible and non-mutually exclusive mechanisms could explain such changes in male socio-sexual behaviors: either they are a by-product of changes in female sexual behaviors (i.e. passive responses to variations in female solicitations), or they are an active response to female signals of pregnancy.

In contrast to previous findings on other macaques species [9, 11], we found that the probability of displaying female sexual behaviors (approaches) significantly decreased from the pre-conceptive period to pregnancy, suggesting that females exhibit behavioral changes during pregnancy that could be used by males as behavioral signals. Our results are in agreement with previous studies showing that captive female Japanese macaque sexual behaviors, including approaches, might vary during the menstrual cycle but do not indicate the fertile period [41, 42]. Taken together, this suggests that female behavioral signals might provide information to males about female reproductive status (cycling vs. pregnant) rather than signaling the precise ovulation window. Using variations in female sexual behaviors between pre- and post-conceptive periods, male Koshima macaques might discriminate pregnancy and decrease their mating behaviors (e.g. male approaches, mounts) toward pregnant females. Simultaneously, our data can’t rule out the possibility that female solicitations are the driving factor influencing male mating-related behaviors. The decrease in sexual initiations from pregnant females during the mating season might therefore induce a decrease in male sexual motivation toward these females, with males focusing their reproductive effort on females who are still cycling and soliciting them at this period.

Changes in face color might be one of the gestational signals available from a distance to all males in order to discriminate the pregnancy. Visual signals, such as the expression of face color, are linked to estrogen concentrations and hence to the timing of ovulation (rhesus macaques: [28, 50, 62], Japanese macaques: [61]), and have been suggested to provide information about conception (rhesus macaques: [63], mandrills: [64]). Using accurate and objective measures of color and luminance, we found that female Japanese macaques undergo changes in facial luminance between pre- and post-conceptive periods, as well as chromatic changes during pregnancy. Skin luminance is linked to concentrations of circulating estrogens, which bind to estrogen receptors in the facial skin causing an increase in blood flow [62, 65]. In our study we could not statistically analyze changes in sexual steroid concentrations between pre- and post-conceptive phases; however Fujita et al. [54] previously reported that estrogen concentrations increased from conception to early pregnancy in female Japanese macaques. It is possible that changes in the ratio of circulating female sexual steroids between pre- and post-conceptive periods might have a greater impact on the amount of sexual skin blood flow, and thus on female face darkness, than on color *per se*. As previously reported by Pflüger et al. [66], changes in female face color seem to elicit both behavioral and endocrinological responses from male Japanese macaques. Thus, it is possible that males might discriminate pregnancy by detecting changes in female face darkness and then modulate their post-conception mating choices. Female facial redness varied solely during pregnancy, with females becoming less red throughout pregnancy. Unlike achromatic variation (strongly linked to blood flow), chromatic variation tends usually to be linked more to blood oxygenation. Chromatic variation may also be more condition-dependent than achromatic variation [62, 67], and it is possible that early changes in energy balance during pregnancy, e.g. allocation of resources to fetal growth, might therefore have an impact on female facial redness.

Auditory signals, i.e. copulation calls, were previously found to influence female and male mating choices in macaques [52] and baboons [68] and to modulate mating outcomes [69, 70]. Yet little is known about the role of female estrus calls in sexual signaling as most studies have focused on copulation calls. In a previous study of Japanese macaques [41], estrus calls varied across the cycle with higher frequencies during the follicular and peri-ovulatory phases compared to the luteal phase. We found that although the intensity of auditory signals (i.e. count of estrus calls) did not vary according to reproductive status, the probability of producing estrus calls significantly decreased from the pre-conceptive period to the first month of pregnancy. No differences were found between the pre-conception period and the second month of pregnancy. The decrease in auditory signals during the first month of pregnancy suggests that estrus calls might convey information about the female reproductive status. However, without data on the acoustical parameters of the calls (e.g. frequency, intensity), it is still too preliminary to draft strong conclusions about the role of estrus calls in Japanese macaque pregnancy signaling. Some auditory signals (e.g. copulation calls) have been found to change in duration and frequency in association with variations in female sexual steroids between pre- and post-conceptive periods (e.g. Barbary macaques: [25]). The acoustic properties of estrus calls in Japanese macaques might undergo structural changes during pregnancy that are discernible to males, which might allow them to discriminate pregnant females. It is also possible that other social events unrelated to mating might have an impact on the probability that auditory signals occur (e.g. female sexual competition, affiliative interactions), leading to an increase during the second month of pregnancy. More data are needed to fully understand the role of auditory signals on Japanese macaque mating strategies.

We cannot rule out the possibility that males might also have access to more subtle cues such as chemical signals. Olfactory cues such as changes in vaginal secretion compounds have been described to contain potentially reliable information about the probability of ovulation (humans: [32], baboons: [29, 47]) and to influence male primate sexual behaviors and mating strategies (stump-tailed macaques: [31], humans: [71], marmosets: [72]). In a recent paper, Crawford and Drea [73] reported that the composition of odorant compounds of vaginal secretions varied from pre-conceptive to pregnancy periods in lemurs, providing potential cues of female reproductive status to males. In our study we did not observe any male olfactory (nasal) inspections of the ano-genital area, but the lack of inspection does not necessarily indicate that females do not emit odorant cues of the reproductive state that males can detect. For instance, we noticed that females who were in consortship and who received copulations emitted a particular strong odor (L. Rigaill personal observation), which might be derived from vaginal secretions and/or from sperm and sperm plugs.

Our findings suggest that female Japanese macaques might signal their pregnancy through changes in sexual signals between pre- and post-conceptive periods, which could be used by males in their mating-decisions. It has been previously suggested that in some populations of Japanese macaques living in harsh environments (i.e. Koshima and Kinkazan Island) there might be a strong selective pressure on both males and females to avoid mating during post-conceptive periods [40]. In this seasonal species, males and females tend to mate and conceive during the coldest months of winter [74], which are also associated with the lowest food availability and variety for island populations [75–78]. It has been previously reported that female Japanese macaques undergo relatively high energetic costs (e.g. thermoregulation and reproductive costs) during the mating season (in captivity, see [79]). Therefore, it is possible that a multimodal system of gestational signaling has merged in some populations of Japanese macaques living in challenging environments in order to avoid wasting energy on non-reproductive mating. The cost of gestational signaling for females might be compensated by immediate benefits, such as decreasing male harassment, and freeing resources to allocate to fetal growth. Simultaneously, males gain direct benefits from pregnancy discrimination, by not wasting energy in sperm production or actively mate guarding pregnant females. It is also important to note that infanticide is very rare in Japanese macaques with to date only one case reported [80], whereas it is not rare in most of the primate species that show post-conceptive sexual behaviors. Thus, pregnancy advertisement might be an evolved strategy of some seasonal primate species living in harsh environments with low infanticide risks compared to those living in less challenging environments [9, 10, 14], for which paternity confusion might drive the evolution of post-conceptive sexual behaviors.

## Conclusion

Our study shows that female Japanese macaques signal their pregnancy through changes in sexual signals such as sexual behaviors, face color parameters and potentially variation in estrus calls (although more data are needed to fully understand the role of acoustic signals in pregnancy signaling). Such changes might modulate male sexual behaviors and motivation to copulate in order to avoid wasting energy on reproductive interactions with females who are already pregnant. By having a signaling system for pregnancy, females and males might both be able to decrease the overall cost of mating, by allocating their energy either to fetal growth for females or to reproductive mating with cycling females for males.

## Supporting Information

S1 File This file contains. **Table A.** Results of likelihood ratio tests (LRT) comparing general linear mixed-effects models (LME) of the face color (N = 88 photos) and hormonal (N = 59 samples) variables.**Table B.** Results of general linear mixed-effects models (LME) examining the relationship between female face color (N = 88 photos), hormonal levels (N = 59 samples) and female reproductive status.**Table C.** Result of likelihood ratio tests (LRT) comparing generalized linear mixed-effects models (GLMM) of behavioral variables (based on N = 153 focal observations).**Table D.** Results of generalized linear mixed-effects models (GLMM) examining the relationship between male/female socio-sexual behaviors (based on N = 153 focal observations) and female reproductive status.
(DOCX)
