# Supplementary material for: Multimodal Advertisement of Pregnancy in Free-Ranging Female Japanese Macaques (Macaca fuscata)
Source: PLoS One. 2015 Aug 26;10(8):e0135127. doi: 10.1371/journal.pone.0135127 (PMC4550261; doi:10.1371/journal.pone.0135127)
Supplement: S1 File — Table A. Results of likelihood ratio tests (LRT) comparing general linear mixed-effects models (LME) of the face color (N = 88 photos) and hormonal (N = 59 samples) variables.Table B. Results of general linear mixed-effects models (LME) examining the relationship between female face color (N = 88 photos), hormonal levels (N = 59 samples) and female reproductive status.Table C. Result of likelihood ratio tests (LRT) comparing generalized linear mixed-effects models (GLMM) of behavioral variables (based on N = 153 focal observations).Table D. Results of generalized linear mixed-effects models (GLMM) examining the relationship between male/female socio-sexual behaviors (based on N = 153 focal observations) and female reproductive status. (DOCX) [file pone.0135127.s001.docx]

Table A. Result of likelihood ratio tests (LRT) comparing generalized linear mixed-effects models (GLMM) of behavioral variables (based on N=153 focal observations). Full models included pregnancy status and age as fixed effects, with date and individual identity made random effects. Null models for comparison included only age as a fixed effect so that we could assess the overall impact of the pregnancy status term in explaining our data. Details of the statistical approach can be found in the main text.

|  | Binary (presence/absence) models | | | | Truncated count models | | | |
| --- | --- | --- | --- | --- | --- | --- | --- | --- |
| **Models** | **Δdf** | **ΔLogLik** | **Chisq** | **P(>Chisq)** | **Δdf** | **ΔLogLik** | **Chisq** | **P(>Chisq)** |
| Female approaches | 4 | 5.288 | 10.575 | **0.005** | 4 | 2.933 | 5.866 | 0.118 |
| Female-directed grooming bouts | 4 | 3.466 | 6.932 | **0.031** | 5 | 0.494 | 0.988 | 0.610 |
| Estrus calls | 4 | 6.666 | 13.333 | **0.001** | 5 | 0.390 | 0.794 | 0.672 |
| Male approaches | x | x | x | x | x | x | x | x |
| Male-directed grooming bouts | 4 | 1.026 | 2.052 | 0.359 | 5 | 0.440 | 0.882 | 0.643 |
| Male holding behaviors^a^ | 3 | 1.723 | 3.446 | 0.063 | x | x | x | x |

^a^ Comparison was possible only between the pre-conceptive period and the second month of pregnancy.

Table B. Results of generalized linear mixed-effects models (GLMM) examining the relationship between male/female socio-sexual behaviors (based on N=153 focal observations) and female reproductive status. Details of the statistical approach can be found in the main text.

|  | **N** | **Factors** | **Est.** | **Std. error** | **z value** | **Pr(>\|z\|)** |
| --- | --- | --- | --- | --- | --- | --- |
| Female approaches | 153 | Intercept | -2.478 | 1.164 | -2.13 | **0.033** |
|  |  | PCP vs. month 1 | 2.124 | 0.774 | -2.75 | **0.006** |
|  |  | PCP vs. month 2 | 1.249 | 0.729 | -1.71 | 0.086 |
|  |  | month 1 vs. 2 | 0.875 | 0.768 | 1.14 | 0.254 |
|  |  | age | -0.035 | 0.086 | -0.41 | 0.683 |
| Female-directed grooming bouts | 153 | Intercept | -4.089 | 1.477 | -2.77 | **0.006** |
|  |  | PCP vs. month 1 | 1.321 | 0.638 | -2.07 | **0.038** |
|  |  | PCP vs. month 2 | 1.445 | 0.689 | -2.10 | **0.036** |
|  |  | month 1 vs. 2 | -0.125 | 0.699 | -0.18 | 0.859 |
|  |  | age | 0.149 | 0.105 | 1.42 | 0.155 |
| Estrus calls | 153 | Intercept | -2.990 | 1.136 | -2.63 | **0.009** |
|  |  | PCP vs. month 1 | 2.230 | 0.698 | -3.19 | **0.001** |
|  |  | PCP vs. month 2 | 0.650 | 0.674 | -0.96 | 0.335 |
|  |  | month 1 vs. 2 | 1.933 | 0.995 | 1.94 | **0.052** |
|  |  | age | 0.051 | 0.081 | 0.63 | 0.526 |
| Male holding behaviors | 153 | Intercept | -2.498 | 0.568 | -4.40 | **< 0.001** |
|  |  | PCP vs. month 1 | x | x | x | x |
|  |  | PCP vs. month 2 | 1.271 | 0.635 | 2.00 | **0.045** |
|  |  | month 1 vs. 2 | x | x | x | x |
|  |  | age | x | x | x | x |

Table C. Results of likelihood ratio tests (LRT) comparing general linear mixed-effects models (LME) of the face color (N=88 photos) and hormonal (N=59 samples) variables. Full models included pregnancy status and age as fixed effects, with date and individual identity made random effects. Null models for comparison included only age as a fixed effect so that we could assess the overall impact of the pregnancy status term in explaining our data. Details of the statistical approach can be found in the main text.

| **Models** | **Δdf** | **Δ\|LogLik\|** | **Chisq** | **P(>Chisq)** |
| --- | --- | --- | --- | --- |
| Luminance | 4 | 3.623 | 7.246 | **0.028** |
| R/G ratio | 4 | 2.329 | 4.658 | 0.097 |
| E1C / PdG Ratio | 4 | 5.797 | 11.593 | **0.001** |
| PdG | 4 | 3.890 | 7.780 | **0.006** |
| E1C | 4 | 0.002 | 0.003 | 0.956 |

Table D. Results of general linear mixed-effects models (LME) examining the relationship between female face color (N=88 photos), hormonal levels (N=59 samples) and female reproductive status. Details of the statistical approach can be found in the main text.

|  | **N** | **Factors** | **Value** | **Std. Error** | **t-value** | **P(>\|t\|)** |
| --- | --- | --- | --- | --- | --- | --- |
| Luminance | 88 | Intercept | -1.994 | 0.165 | -12.11 | 0.000 |
|  |  | PCP vs. month 1 | -0.279 | 0.105 | -2.66 | **0.010** |
|  |  | PCP vs. month 2 | -0.195 | 0.114 | -1.71 | 0.092 |
|  |  | month 1 vs. 2 | 0.084 | 0.108 | 0.77 | 0.441 |
|  |  | age | 0.015 | 0.013 | 1.10 | 0.354 |
| R/G ratio | 88 | Intercept | -2.345 | 0.176 | -13.32 | 0.000 |
|  |  | PCP vs. month 1 | 0.132 | 0.107 | 1.24 | 0.219 |
|  |  | PCP vs. month 2 | -0.102 | 0.116 | -0.87 | 0.384 |
|  |  | month 1 vs. 2 | -0.233 | 0.110 | -2.12 | **0.037** |
|  |  | age | -0.009 | 0.014 | -0.63 | 0.575 |
| E1C / PdG ratio | 59 | Intercept | -2.327 | 0.196 | -11.89 | 0.000 |
|  |  | month 1 vs. 2 | 0.359 | 0.103 | 3.49 | **0.001** |
|  |  | age | -0.012 | 0.016 | -0.78 | 0.494 |
| PdG | 59 | Intercept | -0.023 | 0.326 | -0.07 | 0.945 |
|  |  | month 1 vs. 2 | -0.356 | 0.133 | -2.90 | **0.006** |
|  |  | age | 0.046 | 0.023 | 1.96 | 0.144 |
